# Longitudinal assessment and determinants of short-term and longer-term psychological distress in a sample of healthcare workers during the COVID-19 pandemic in Quebec, Canada

**DOI:** 10.3389/fpsyt.2023.1112184

**Published:** 2023-05-18

**Authors:** Filippo Rapisarda, Nicolas Bergeron, Marie-Michèle Dufour, Stéphane Guay, Steve Geoffrion

**Affiliations:** ^1^Research Centre, Institut universitaire en sant9́ mentale de Montréal (IUSMM), Montreal, QC, Canada; ^2^Département de psychiatrie et d'addictologie, Faculté de Médecine, Université de Montréal, Montreal, QC, Canada; ^3^Research Centre, Centre hospitalier de l'Université de Montréal, Montreal, QC, Canada; ^4^École de psychoéducation, Faculté des arts et des sciences, Université de Montréal, Montreal, QC, Canada; ^5^École de criminologie, Faculté des arts et des sciences, Université de Montréal, Montreal, QC, Canada

**Keywords:** psychological distress, COVID-19, healthcare workers (HCWs), depression, anxiety, post-traumatic stress, risk factors, intensive longitudinal assessment

## Abstract

**Introduction:**

Previous research has demonstrated the negative impact of the COVID-19 pandemic emergency on the wellbeing of healthcare workers. However, few research contributions reported a longitudinal evaluation of psychological distress and examined determinants of its duration and course over time. The present study aims to explore the impact of the pandemic emergency on HCWs mental health by adopting a longitudinal design and assessing mental health as combination of overlapping clinical symptoms (post-traumatic stress disorder, depression and anxiety).

**Methods:**

Data were collected weekly through a mobile application during and after the first wave of COVID-19 in the province of Quebec, Canada, in 2020. Analysis was conducted on a final sample of 382 participants. Participants were grouped into “resilient” (RES) if they did not manifest clinical-level psychological distress during monitoring, “short-term distress” (STD) if distress exceeded the clinical threshold for 1–3 weeks, and longer-term distress (LTD) if it occurred for four or more weeks, even if not consecutively. Descriptive statistics for all variables were computed for each subgroup (RES, STD and LTD), and pairwise comparisons between each group for every descriptive variable were made using chi square statistics for categorical variables and t-test for continuous variables. Predictors of distress groups (STD and LTD vs RES) were assessed running multinomial hierarchical logistic regression models.

**Results:**

In our sample, almost two third (59.4%) HCWs did not manifest moderate or severe distress during the monitoring time. Short-term distress, mostly post-traumatic symptoms that lasted for less than 4 weeks, were the most common distress response, affecting almost one third of participants. Longer psychological distress occurred only in a smaller percentage (12.6%) of cases, as a combination of severe posttraumatic, depressive and anxiety symptoms. Perceived occupational stress was the most significant risk factor; moreover individual, peritraumatic work and family risk and protective factors, were likely to significantly affect the stress response.

**Discussion:**

Results tend to provide a more complex and resiliency-oriented representation of psychological distress compared to previous cross-sectional studies, but are in line with stress response studies. Findings allow us to better describe the profiles of distress response in STD and LTD groups. Participants that manifest short term distress experience acute stress reaction in which the interplay between personal, family and professional life events is associated with the stress response. Conversely, longer term distress response in HCWs presents a more complex mental health condition with an higher level of impairment and support needs compared to participants with short-term distress.

## 1. Introduction

After 2 years into the COVID-19 pandemic, an abundant body of research has demonstrated the negative impact on the wellbeing of healthcare workers (HCWs) in different settings and geographic areas. Psychological distress, commonly defined as a state of emotional suffering characterized by non-specific psychological or somatic symptoms that could either spontaneously resolve or evolve toward a clinical condition (1, 2) has been assessed and estimated in healthcare and social services in different Countries. According to meta-analysis studies (3–5), prevalence of psychological distress symptoms and syndromes in healthcare workers was estimated as follow: depressive symptoms ranged from 31.8% to 60.5%, major depressive disorder 13.4%, anxiety symptoms from 34.2% to 57.7%, anxiety disorders 7.4%; post-traumatic stress symptoms from 21.4% to 65.4%, acute stress disorder 7.4%, and post-traumatic stress disorder (PTSD) 21.7%. Psychological distress could hinder performance in HCWs (6) and may increase rates of sick-leave (7), as already documented during the COVID-19 pandemic (8), furtherly contributing to increase occupational stress related to understaffing. Monitoring HCWs' psychological distress is a strategy for early detection of at-risk workers and may be included in the design of organization-based programs to foster staff wellbeing, resilience and recovery. A public health emergency, like the COVID-19 pandemic, increases the necessity of systematic approach based on research evidence.

However, most of the research conducted over HCWs has some methodological limitations that may hinder its applicative relevance in the field of occupational mental health prevention and clinical management. The most frequent is the use of a cross-sectional design that assess individual status only at the time the data are collected, usually looking back in the last 1 or 2 weeks, as requested in most self-administered questionnaires. This design is not suitable for detecting intra-individual change across time (9) and provides a static representation of psychological distress. In this way, it is not possible to determine whether, for example, a certain level of detected distress (e.g., depressive symptoms) is transient and likely to resolve spontaneously or constitutes a longer-lasting mental health problem with a greater impact on job performance and wellbeing. Conversely, a limited number of longitudinal studies applied insights from the posttraumatic trajectory research (10) to examinate stress responses in the general population (11–13) and few on HCWs (14, 15) during the pandemic. The resilient trajectory was detected in most of the participant, while some other experienced short term, sub-chronic or delayed distress response. Moreover, in trajectory studies based on exploratory statistical modeling (like latent class analysis), the shape and width of the estimated curves depend on the length of the observation period and the distribution of values in the sample studied. At the same time, the aforementioned studies could provide a rationale for adopting an operationalization of distress response based on its duration, and could provide useful application insights for clinical practice, mental health assessment in work settings, or even the development of digital apps and tools for individual wellbeing.

Another relevant issue concerns the simultaneous use of different symptom scales. Most studies assessed psychological distress using a combination of self-administered scales, usually one for depressive symptoms in combination with others that assess, depending on the study, anxiety symptoms, burnout, insomnia, PTSD. However, few studies estimate the co-presence of symptoms collected from different scales, preferring to estimate separately, for example, the prevalence of moderate or severe anxiety symptoms and the prevalence of depressive symptoms. However, research suggests that overlap between symptoms of anxiety, depression and or PTSD is frequent (16–19) during clinical assessment and in psychometric analysis using factor analytic techniques and network analysis approaches (20, 21). There is also an ongoing theoretical debate with respect to explaining this overlap as a comorbidity (between depression and PTSD) or a specific subtype of PTSD (22).

Therefore, the purpose of the present study is to contribute to increase the knowledge of psychological distress reaction in occupational settings to design new studies and occupational health practices by better depicting profiles of HCWs based on the longitudinal course of distress and its determinants. For this scope, we adopted specific methodological approaches that could overcome the aforementioned issues, i.e., (1) an intensive longitudinal assessment design, collecting data on a weekly base; (2) an overall index of psychological distress obtained by combining different scales; (3) applying a simple classification of distress profiles based on distress duration to obtain clinically relevant subgroups; (4) comparing lifetime, clinical and work related characteristics and risk factors between the different subgroups to generate insights.

In particular, the main novelty introduced in the study is the use of a 4-week threshold to differentiate the profiles of HCWs experiencing moderate and severe distress. Previous studies already adopted clinical cutoffs to estimate individuals with clinically relevant psychological distress leves. We propose to furtherly cluster non-resilient individuals in two subgroups, i.e., participants who scored above the clinical cut of in one or more measures for 1–3 weeks, experiencing a short-term distress (STD); longer-term distress (LTD) group, grouping together participants that experience clinical distress for 4 weeks or more. The adoption of a 4 week period of clinical distress to differentiate between STD and LTD groups is an attempt to operationalize findings from previous studies and clinical guidelines. In fact, previous studies (14, 15) that reported a differentiation of short (recovered trajectory) and long term (sub-chronic trajectory) distress trajectories after the first month. Our definition of STD would be similar to “recovered” profile in trajectory studies and clinically could be defined as transient stress reactions. Conversely, the LTD group could encompass those cases that trajectory studies identify as “subchronical” or as even subjects with possible PTSD; in fact, according to United Kingdom's National Health Service, 1 month of post-traumatic symptoms is required for a diagnosis of PTSD (23).

Thus, a first objective is to explore quantitative and qualitative differences between short term and longer term distress groups and a second objective is the search for determinants of different profiles of clinical symptoms.

## 2. Methods

### 2.1. Design and sample

This prospective cohort study collected data through a mobile application during and after the first wave of COVID-19 in the province of Quebec, Canada, between May 8, 2020, and January 24, 2021. Preliminary findings, using latent class analysis, were reported in previous papers (14, 15). The design merged the classical principles of prospective cohort studies with some methodological aspects of the Ecological Momentary Assessment (EMA) methodology (24): participants were asked to fill several questionnaires through the mobile application on a weekly basis to collect data on both distress trends and possible associated factors, such as perceived occupational stress, family support or adverse experiences. Compared to retrospective cross-sectional methods, EMA is a self-report data collection method that may reduce recall bias (25). Moreover, compared to pulse-surveys, EMA studies use a more limited set of variables collected over several close assessment times to identify the cause/effect relationship between variables over time or the existence of trajectories.

EMA data collection was anonymous, confidential, and on a voluntary basis. The research ethics board of the University of Montreal Hospital Research Center approved the research project (project number: MP-02-2021-8963, 20.015). Written consent of every participant was obtained before their participation. Eight health-care institutions in the province of Quebec participated in the study. The research team began by contacting research coordinators in every clinical setting. The communication services then distributed promotional material through various platforms to reach all employees. Interested HCWs transmitted their consent (either through a web form or by directly emailing the research coordinator). After the reception of the consent form, each participant received instructions for installing the mobile application. Once the participant launched the app, he or she received an user Id, so investigators cannot link responses to participants identities. Eight hundred and thirty-two HWCs registered in the monitoring app and were enrolled in the study.

### 2.2. Data collection and instruments

Data were collected using two different collection strategies: on weekly basis, a mobile application, Ethica (https://ethicadata.com/), presented self-monitoring questions and items about potential source of distress and support occurred during the week; at the end of the monitoring period, every registered participant received a link to an online survey through the SurveyMonkey platform (https://www.surveymonkey.com/) that collected, retrospectively, potential proximal and distal risk and protective factors.

#### 2.2.1. Psychological distress

Psychological distress was assessed weekly through the French versions of the following validated instruments that have been widely used in population studies conducted during the pandemic in several countries: the short version of Post-Traumatic Stress Disorder Checklist for Diagnostic and Statistical Manual of Mental Disorders, fifth edition (PCL5-8); the General Anxiety Disorder-7 (7 items; GAD-7), and the Patient Health Questionnaire (9 items; PHQ-9). The GAD-7 (26, 27) was used to assess symptoms of anxiety and as indicator of level of psychological distress. It has been the most frequent used instrument to assess anxiety levels in the general population and also among healthcare workers during the COVID-19 pandemic. The PHQ-9 (28) is a validated questionnaire that assesses the presence of depressive symptoms among patients and it has been frequently adopted to assess psychological distress in the general population and among healthcare workers during the COVID-19 pandemic. In our sample, internal consistency was good for GAD-7 (Cronbach's alpha = 0.90) and PHQ-9 (Cronbach's alpha = 0.87). The PCL5-8 has been developed to screen for posttraumatic symptomatology. The “global score version” of the 8-item scale was adopted (29) to use it like a screening tool in the same way of the PHQ9 and GAD7. The resulting scale had a good internal consistency (Cronbach's alpha = 0.90). The results were interpreted according to the following clinical cut-off scores: 13 for PCL-5, 10 for GAD-7 and for PHQ-9. For each measure, the look-back period was 7 days, instead of the commonly adopted 2-week period, as the participants were invited to fill out questionnaires every week.

#### 2.2.2. Determinants of psychological distress

Based on the literature of psychological distress in HCWs and during disasters, a selection of variables believed to be possible determinants of distress were collected to test a predictive model. Variables were grouped in three conceptual levels. First, personal vulnerability risk factors, i.e., the presence of a lifetime mental health diagnosis, retrieved using *ad hoc* items, and lifetime occurrence of stressful and traumatic events that were collected using the Life Events Checklist (LEC-5) (30); biological sex and age were also included in that group. Lifetime mental health problems and adverse events have been indicated as possible risk factors for the development of depressive disorder or PTSD in HCWs during the pandemic (31).

The second group of variables comprehended the work-related variables. Participants indicated if they were working in units that could be associated with increased COVID-19 exposition, such as emergency ward, intensive care unit, nursing home, or COVID-19 ward. On a weekly basis, their level of COVID-19 related fear at work was assessed. Findings from previous research indicates that direct exposition to COVID-19 patients in emergency wards or at-risk units increased the fear of COVID-19 infection which, in turn, negatively impacted on emotional exhaustion and psychological distress (32–37). Stressful events related to work environment, that have been documented in previous studies (32, 34, 38, 39), i.e., personal protective equipment (PPE) shortage, lack of personnel, procedure-challenging restrictions, COVID-19 outbreak at the unit. Since witnessing patients' negative experiences and deaths during an emergency or disaster like context could elicit posttraumatic distress (40, 41), COVID-19-related deaths of colleague or patients were registered. Perceived stress (42) level at work was assessed weekly, on a scale that ranged from 0 (not stress at all) to 10 (very high stress). Perceived stress has been associated with PTSD in HCWs during the pandemic (43, 44). The pandemic emergency required services to modify procedures, most of which were related to sanitation and infection control, and redeploying personnel in a relatively short time. Consequently, the rise in workload and the adjustment to new procedures increased the occupational strain (33, 34, 38, 39, 45–47) that negatively impacts on HCWs mental health. Two items measured perceived availability of social support from colleagues and the organization on a scale 4 point scale from 0 (source of support never available) to (source of support always available). Perceived lack of organizational support was also associated with increased burnout exhaustion and psychological distress (32, 46) and, conversely, social support from colleagues (47) could moderate the effect of risk factor over distress.

The third level grouped personal and family life factors. Family-related stressors and events comprehended: the death of a family member; living with children and taking care of another family member (elderly or person with disabilities) that could have raised distress and strain by increased the fear of contagion and work-family balance. Personal life factors included: being quarantined, that could be associated to a perception of threat and rupture of social contacts, being positive to COVID-19 test, that could be experienced as a traumatic event and being vulnerable to COVID-19 for any medical reasons, that could rise fear of contagion. Moreover, perceived availability of social support from family members was measured as already described for colleagues and the organization. Finding from previous studies indicated that social support and emotional connectedness from family members could have a protective effect on HCWs mental health (47, 48).

To summarize, determinants were collected in two different phases: (a) determinants that were collected repeatedly during the monitoring phase every week though the app: weeks of perceived high occupational stress, fear of COVID-19, perceived availability of support from colleagues, from the organization and from relatives; (b) determinants that were collected retrospectively at the end of the monitoring phase: some were related to lifetime participants' characteristics: biological sex, age, lifetime diagnosis of mental health problems, adverse events lifetime (LEC-5), and some others depicted events that occurred during the monitoring phase: working in an at-risk unit (emergency unit, Intensive care, COVID-19 ward, etc…), PPE shortage, lack of personnel, work-challenging restrictions, COVID outbreak in the work unit, death of colleagues or more patients due to COVID-19, infants at home, caregiving of a family member, being vulnerable to COVID-19, being quarantined, being positive to COVID-19 test, COVID-19-related death of family member and loss of income.

### 2.3. Data analysis

Participants were included in the analysis if filled out the distress questionnaires at least 6 times in the 8-week period. Four hundred and ten participants didn't fill in the post-test retrospective questionnaire and were excluded from the dataset and 80 out of 460 participants stopped monitoring before week 7 and were excluded from analysis ad dropouts even though they filled in the post test questionnaire. Analysis was conducted on a sample of 382 participants across 8 weeks of monitoring, and occasional missing distress data (occurred in < 15% of the overall data points) were imputed using last observation carried forward (LOCF) method. The LOCF replacement was chosen because of the repeated measure design of the data collected, in which was assumed that the values from the previous week may recur in the next week as well. Before proceeding with the analyses, a preliminary comparison was made between the prevalence of distress groups (RES, STD and LTD) obtained in one LOCF dataset vs. another in which cases with missing data were eliminated listwise: since similar results were obtained, it was decided to continue with the LOCF dataset.

Descriptive statistics for all variables were computed for each subgroup (RES, STD and LTD), and pairwise comparisons between each group for every descriptive variable were made using chi square statistics for categorical variables and *t*-test for continuous variables.

Predictors of distress groups (STD and LTD vs. RES) were assessed running a multinomial hierarchical logistic regression model using VGLM package in R. Predictor variables were introduced in the model in blocks, to better depict each variable effect. The fist block consisted in personal vulnerability risk factors, such as biological sex, age, lifetime diagnosis of mental health conditions and LEC-5 scores. Block two comprised work-related factors. Block three introduced personal and family life factors. Nagelkerke's Pseudo R-squared was computed at each step to estimate the proportion of the total variation of the dependent variable can be explained by independent variables in the current model.

## 3. Results

Most of the participants were female (*n* = 334; 87.4%) with a mean age of 40.6 years (sd = 9.8). Approximately one out of three participants *n* = 192, 31.2%) declared to work in one or more critical setting due to the COVID pandemic, such as a COVID Ward (*n* = 63, 16.5%), an elderly care unit (*n* = 43, 11.3%), an emergency care unit (*n* = 27, 7.1%) or an intensive care unit (*n* = 23, 6%). Regarding lifetime clinical variables, 18.6% of the participants reported a lifetime depression, 16.2% an anxiety disorder, 6.8% a PTSD, and the average number lifetime traumatic/stressful events (LEC5), was 3.2 (sd = 2.5). Scores of psychological distress questionnaire at baseline indicated that mean PHQ-9 score was 6.4 (sd = 4.8), with 12% of with a moderate or severe depression, PCL-5 average score was 7.3 (sd = 5.7), with 17% with a clinical level of PTSD symptoms, and an average GAD-7 score of 5.9 (sd = 4.3), with 9.3% of the sample with moderate or severe anxiety.

Two hundred and two participants (53.2%) did not score on clinical questionnaires above the threshold in the 8 weeks of monitoring and were classified as resilient (RES); 131 (34.3%) exceeded the clinical threshold for <4 weeks (average 1.7) and were classified as short-term distress (STD); 49 (12.8%) showed signs of distress above the threshold for 4 or more weeks and were classified as longer-term distress (LTD).

### 3.1. Comparison between subgroups

Tables 1, 2 presents pairwise comparisons between the three groups. Compared to resilient group, participant classified as STD or LTD reported an higher prevalence of anxiety problems lifetime, higher levels of work-related stress and fear of catching COVID-19 at work, more frequent work-challenging restriction and lower levels of support from the organization, and from family members, experienced quarantined more frequently during the monitoring time, received professional support in the community and through employee assistance programs. At the same time, the STD and LTD groups showed differences. When compared to resilient ones, participants in the STD group were significantly younger, in the work setting experienced more frequently lack of personnel and COVID-19-related deaths of patients, and in the family-life domain reported more frequently having children at home and taking care of other members. Moreover, compared to the RES and STD group, participants in LTD group reported higher prevalence of depression, PTSD and adverse events (LEC-5) lifetime, higher levels of occupational stress and lower support from colleagues and relatives, received more professional help, and reported more sick leaves for physical and psychological reasons.

**Table 1:** Bivariate comparison of work related and personal related characteristics of clinical, short-term and longer-term symptoms subgroups.

	**RES *N* = 223**	**STD** ***N*** = **111**		**LTD** ***N*** = **48**		
			**p**_1_		**p**_1_	**p**_2_
**Personal vulnerability factors**						
Biological sex, female	191 (85.7%)	102 (91.9%)		41 (85.4%)		
Age, mean (sd)	41.7 (10.6)	38.9 (9.6)	^*^	39.9 (8.8)		
Lifetime diagnosis of depression	30 (13.5%)	22 (19.8%)		19 (39.6%)	^***^	^***^
Lifetime diagnosis of anxiety	26 (11.7%)	25 (11.5%)	^**^	11 (22.9%)	^*^	
Lifetime diagnosis of ptsd	11 (4.9%)	7 (6.3%)		8 (16.7%)	^**^	^*^
Adverse events lifetime (LEC-5)	3.0 (2.2)	3.1 (2.8)		4.3 (2.8)	^***^	^*^
**Work-related variables**						
Working in an emergency unit	19 (8.5%)	6 (5.4%)		2 (4.2%)		
Working in an intensive care	11 (4.9%)	8 (7.2%)		4 (8.3%)		
Working in an elderly care unit	20 (9.0%)	15 (13.5%)		8 (16.7%)		
Working in an COVID-19 ward	42 (18.8%)	14 (12.6%)		7 (14.4%)		
Redeployed in a new unit	55 (24.7%)	38 (34.2%)	.	17 (35.4%)		
Weeks of perceived high occupational stress, mean (sd)^a^	2.4 (2.5)	3.0 (2.4)	^*^	4.4 (2.5)	^***^	^*^
Fear of COVID-19, mean (sd)^b^	3.6 (2.2)	3.8 (2.0)	^*^	4.3 (2.0)	^*^	
Perceived availability of support from colleagues^c^	4.5 (3.1)	4.3 (2.8)		2.8 (2.8)	^***^	^**^
Perceived availability of organizational support^c^	3.1 (3.1)	1.9 (2.8)	^***^	1.3 (2.2)	^***^	
PPE shortage	46 (20.6%)	24 (21.6%)		17 (35.4%)	^*^	.
Lack of personnel	88 (39.5%)	57 (51.4%)	^*^	24 (50.0%)		
Work-challenging restrictions	88 (37.2%)	61 (55.0%)	^**^	31 (64.6%)	^***^	
COVID outbreak in the work unit	44 (19.7%)	31 (27.9%)	.	16 (33.3%)	^*^	
Death of colleague due to COVID-19	6 (2.7%)	2 (1.8%)		1 (2.1%)		
Death of one or more patients due to COVID-19	28 (12.6%)	24 (21.6%)	^*^	11 (22.9%)	.	
**Personal and family life variables**						
Perceived availably of support from relatives, mean (sd)^c^	5.4 (3.0)	4.4 (3.0)	^**^	4.1 (3.0)	^**^	
Infants at home	38 (17.0%)	35 (31.5%)	^**^	11 (22.9%)		
Caregiving of a family member	21 (9.4%)	22 (19.8%)	^**^	6 (12.5%)		
Vulnerable to COVID-19	51 (22.9%)	22 (18.8%)		12 (25.0%)		
Quarantined	34 (14.3%)	24 (21.6%)	^*^	15 (31.2%)	^**^	
Positive to COVID-19 test	18 (8.1%)	7 (6.3%)		3 (6.2%)		
COVID-19-related death of family member	4 (1.8%)	5 (4.5%)		2 (4.2%)		
Loss of income	13 (5.8%)	4 (4.6%)		2 (4.2%)		

Study: Longitudinal assessment and determinants of short-term and longer-term psychological distress in a sample of healthcare workers during the COVID-19 pandemic in Quebec, Canada, 2020–2021.

STD, short-term distress; LTD, longer-term distress; p1, comparison vs. resilient group; p2, comparison vs. short-term symptoms group; PPE, Personal Protective Equipment; ^a^Count of weeks in which occupational stress was reported as moderate or high; ^b^Average value of fear across 8 weeks; ^c^Count of weeks in which the source of support was reported as available most of the time; ^**.**^p < 0.10, ^*^p < 0.05; ^**^p < 0.01; ^***^p < 0.001.

**Table 2:** Comparison of clinical characteristics of resilient, short term and longer-term symptoms subgroups.

	**RES**	**STD**		**LTD**		
			**p**_1_		**p**_1_	**p**_2_
**Symptoms assessment**						
**Post-traumatic symptoms (PCL5)**						
Prevalence of moderate/sever symptoms	n.a.	90 (81.1%)	n.a.	40 (83.7%)	n.a.	
Weeks of moderate/severe symptoms, mean (sd)	n.a.	1.3 (1.6)	n.a.	3.5 (2.6)	n.a.	^**^
**Depressive symptoms (PHQ-9)**						
Prevalence of moderate/sever symptoms	n.a.	48 (43.2%)	n.a.	39 (81.2%)	n.a.	^**^
Weeks of moderate/severe symptoms, mean (sd)	n.a.	0.8 (1.1)	n.a.	3.4 (2.8)	n.a.	^**^
**Anxiety symptoms (GAD-7)**						
Prevalence of moderate/sever symptoms	n.a.	46 (41.4%)	n.a.	39 (81.2%)	n.a.	^**^
Weeks of moderate/severe symptoms, mean (sd)	n.a.	0.5 (0.9)	n.a.	2.5 (2.4)	n.a.	^**^
Weeks with any psychological distress, mean (sd)	n.a.	2.1 (1.5)	n.a.	5.7 (1.4)	n.a.	^**^
**Resources for distress management**						
Self-care (healthy living, breathing exercises, etc.)	105 (47.1%)	56 (50.5%)		28 (58.3%)		
Professional help in the community	18 (8.1%)	23 (20.7%)	^***^	19 (39.6%)	^***^	^*^
Employee assistance program (EAP)	14 (6.3%)	17 (15.3%)	^**^	13 (27.1%)	^***^	.
**Sick leave during the study period**						
Sick leave due to physical needs/illness	27 (12.1%)	19 (17.1%)		17 (35.4%)	^***^	^*^
Sick leave due to psychological needs	16 (7.2%)	17 (15.3%)	^*^	21 (43.8%)	^***^	^***^

Study: Longitudinal assessment and determinants of short-term and longer-term psychological distress in a sample of healthcare workers during the COVID-19 pandemic in Quebec, Canada, 2020–2021.

STD, short-term distress; LTD, longer term distress; p1, comparison vs. resilient group; p2, comparison vs. short-term symptoms group; PCL-5, Post-traumatic Stress Disorder Checklist; GAD-7, Generalized Anxiety Disorder Questionnaire; PHQ-9, Patient Health Questionnaire; LEC-5, Life Events Checklist; ^.^p < 0.10, ^*^p < 0.05; ^**^p < 0.01; ^***^p < 0.001; n.a., not applicable.

### 3.2. Determinants of distress levels

**Figure 1:**
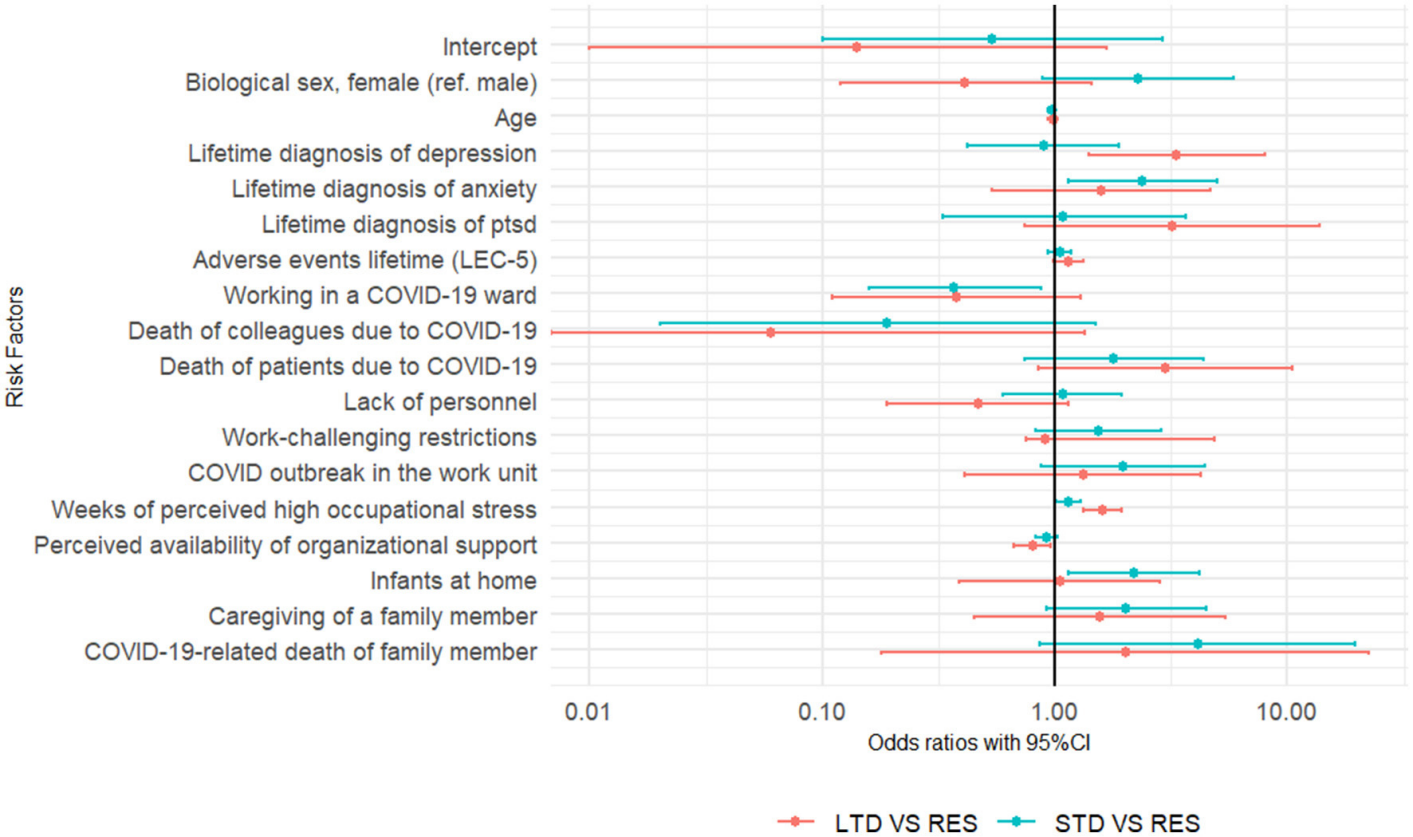
Graphical plotting of risk factors' odds ratios with 95% confidence intervals for short term distress (STD) vs. resilient (RES) and longer-term distress (LTD) vs. RES. Study: Longitudinal assessment and determinants of short-term and longer-term psychological distress in a sample of healthcare workers during the COVID-19 pandemic in Quebec, Canada, 2020–2021.

Table 3 and Figure 1 presents results of the hierarchical multinomial logistic regression. Low to moderate levels of collinearity were assessed and evaluated acceptable for the analysis. In the final model, lower age (OR = 0.97, *p* < 0.05) and lifetime diagnosis of anxiety (OR = 2.93, *p* < 0.05) increased the odds of STD groups compared to RES, and a lifetime diagnosis of depression (OR = 3.35, *p* < 0.01) increased the risk for both distress groups. A subthreshold effect of biological sex (OR = 2.28, *p* < 0.09) was also detected with STD. A significant effect of adverse lifetime events (OR = 1.16, *p* < 0.05) was detected for the LTD group in the Step 1, but the effect became subthreshold (OR = 1.14, *p* < 0.09) when proximal variables are introduced into the model. Work related proximal variables explained most of the variance in the final model (29% over 39% according to Nagelkerke's Pseudo-*R*^2^). More in detail, in the final model weeks of perceived high occupational stress increased the odds of STD (OR = 1.14, *p* < 0.05) and LTD (OR = 1.60, *p* < 0.001) groups, working in a COVID-19 unit reduced the odds of short-term distress (OR = 0.37, *p* < 0.05) and perceived organizational support reduced the risk (OR = 0.80, *p* < 0.05) of longer-term distress. For LTD, subthreshold effects were detected for work-related variables, i.e., COVID-19-related death of a colleague (OR = 0.06, *p* < 0.08) and patients (OR = 2.99, *p* < 0.09), and lack of personnel (OR = 0.47, *p* < 0.1). Concerning the family and personal proximal variables, having children at home (OR = 2.19, *p* < 0.05) increased the odds of STD; a subthreshold effect of taking care of other family member (OR = 2.02, *p* < 0.08) and COVID-19-related death of family member (OR = 4.12, *p* < 0.08) were also detected.

**Table 3:** Assessing determinants of short-term or longer-term psychological distress using hierarchical multinomial regression.

	**Short term distress vs. resilient**			**Longer term distress vs. resilient**		
	***B* (SE)**	**OR (95%CI)**	**p**	***B* (SE)**	**OR (95%CI)**	**p**
**Block 1: personal vulnerability factors (Pseudo-***R*^2^ = **0.12)**						
Intercept	−0.35 (0.62)	0.7 (0.21–2.38)		−1.39 (0.83)	0.25 (0.05–1.26)	.
Biological sex, female (ref. male)	0.64 (0.41)	1.9 (0.86–4.22)		−0.21 (0.48)	0.81 (0.32–2.07)	
Age	−0.03 (0.01)	0.97 (0.95–0.99)	^*^	−0.02 (0.02)	0.98 (0.95–1.01)	
Lifetime diagnosis of depression	0.27 (0.33)	1.3 (0.69–2.47)		1.25 (0.38)	3.48 (1.64–7.38)	^**^
Lifetime diagnosis of anxiety	0.65 (0.32)	1.92 (1.03–3.61)	^*^	0.23 (0.46)	1.26 (0.52–3.1)	
Lifetime diagnosis of ptsd	−0.05 (0.52)	0.95 (0.34–2.66)		0.81 (0.56)	2.26 (0.76–6.72)	
Adverse events lifetime (LEC-5)	0.04 (0.05)	1.04 (0.94–1.14)		0.15 (0.06)	1.16 (1.02–1.31)	^*^
**Block 2: work-related factors (Pseudo-***R*^2^ = **0.35)**						
Intercept	−0.86 (0.78)	0.43 (0.09–1.97)		−2 (1.21)	0.14 (0.01–1.44)	.
Biological sex, female (ref. male)	0.6 (0.45)	1.82 (0.75–4.41)		−0.7 (0.62)	0.5 (0.15–1.69)	
Age	−0.03 (0.01)	0.97 (0.95–1)	.	−0.02 (0.02)	0.98 (0.94–1.03)	
Lifetime diagnosis of depression	0 (0.36)	1 (0.49–2.02)		1.21 (0.44)	3.37 (1.43–7.92)	^**^
Lifetime diagnosis of anxiety	0.96 (0.36)	2.6 (1.3–5.22)	^**^	0.58 (0.54)	1.79 (0.62–5.14)	
Lifetime diagnosis of ptsd	0.09 (0.58)	1.09 (0.35–3.43)		1.48 (0.71)	4.39 (1.09–17.7)	^*^
Adverse events lifetime (LEC-5)	0.05 (0.06)	1.06 (0.95–1.18)		0.11 (0.07)	1.12 (0.97–1.28)	
Working in a COVID-19 ward	−0.9 (0.41)	0.41 (0.18–0.9)	^*^	−1.1 (0.61)	0.33 (0.1–1.1)	.
Death of colleague due to COVID-19	−1.25 (0.97)	0.29 (0.04–1.93)		−2.62 (1.46)	0.07 (0–1.27)	.
Death of one or more patients due to COVID-19	0.69 (0.43)	1.99 (0.85–4.64)		1.17 (0.64)	3.23 (0.92–11.31)	.
Lack of personnel	0.14 (0.29)	1.15 (0.66–2.02)		−0.78 (0.44)	0.46 (0.19–1.09)	.
Work-challenging restrictions	0.53 (0.3)	1.7 (0.94–3.07)	.	0.64 (0.46)	1.9 (0.78–4.67)	
COVID outbreak in the work unit	0.48 (0.39)	1.61 (0.76–3.44)		0.13 (0.57)	1.14 (0.38–3.45)	
Weeks of perceived high occupational stress	0.13 (0.06)	1.14 (1.01–1.29)	^*^	0.47 (0.09)	1.6 (1.33–1.92)	^***^
Perceived availability of organizational support	−0.15 (0.05)	0.86 (0.77–0.96)	^**^	−0.19 (0.09)	0.82 (0.69–0.99)	^*^
**Block 3: personal and family factors (Pseudo-***R*^2^ = **0.39)**						
Intercept	−0.62 (0.86)	0.54 (0.1–2.91)		−1.96 (1.26)	0.14 (0.01–1.66)	
Biological sex, female (ref. male)	0.82 (0.49)	2.28 (0.88–5.91)	.	−0.89 (0.64)	0.41 (0.12–1.44)	
Age	−0.03 (0.01)	0.97 (0.94–1)	^*^	−0.01 (0.02)	0.99 (0.94–1.03)	
Lifetime diagnosis of depression	−0.11 (0.38)	0.9 (0.42–1.9)		1.21 (0.44)	3.35 (1.4–8.01)	^**^
Lifetime diagnosis of anxiety	0.87 (0.38)	2.39 (1.14–5)	^*^	0.46 (0.55)	1.58 (0.54–4.67)	
Lifetime diagnosis of ptsd	0.09 (0.61)	1.09 (0.33–3.64)		1.16 (0.75)	3.19 (0.74–13.73)	
Adverse events lifetime (LEC-5)	0.05 (0.06)	1.06 (0.94–1.18)		0.13 (0.08)	1.14 (0.98–1.32)	.
Working in a COVID-19 ward	−0.99 (0.43)	0.37 (0.16–0.87)	^*^	−0.96 (0.63)	0.38 (0.11–1.3)	
Death of colleague due to COVID-19	−1.66 (1.06)	0.19 (0.02–1.51)		−2.88 (1.62)	0.06 (0–1.35)	.
Death of one or more patients due to COVID-19	0.59 (0.45)	1.8 (0.74–4.35)		1.1 (0.64)	2.99 (0.85–10.58)	.
Lack of personnel	0.08 (0.3)	1.08 (0.6–1.95)		−0.76 (0.45)	0.47 (0.19–1.14)	.
Work-challenging restrictions	0.44 (0.31)	1.55 (0.83–2.86)		0.65 (0.48)	1.91 (0.75–4.88)	
COVID outbreak in the work unit	0.67 (0.41)	1.96 (0.87–4.42)		0.28 (0.59)	1.32 (0.41–4.25)	
Weeks of perceived high occupational stress	0.13 (0.06)	1.14 (1.01–1.29)	^*^	0.47 (0.1)	1.6 (1.33–1.93)	^***^
Perceived availability of organizational support	−0.08 (0.05)	0.92 (0.83–1.03)		−0.22 (0.09)	0.8 (0.67–0.96)	^*^
Infants at home	0.78 (0.33)	2.19 (1.15–4.17)	^*^	0.05 (0.5)	1.05 (0.39–2.82)	
Caregiving of a family member	0.71 (0.4)	2.02 (0.92–4.47)	.	0.44 (0.64)	1.56 (0.45–5.45)	
COVID-19-related death of family member	1.42 (0.8)	4.12 (0.86–19.6)	.	0.7 (1.23)	2.02 (0.18–22.42)	

Study: Longitudinal assessment and determinants of short-term and longer-term psychological distress in a sample of healthcare workers during the COVID-19 pandemic in Quebec, Canada, 2020–2021.

OR, odds ratio; ^.^p < 0.10, ^*^p < 0.05; ^**^p < 0.01; ^***^p < 0.001; the following variables were included in the models but are not displayed because no significant effects were found: lack of PPE, loss of income, vulnerable to COVID-19, quarantined, positive to COVID-19 test, COVID-19-related death of family member, perceived support from family members.

## 4. Discussion

The present study attempted to depict HCWs distress response (and its determinants) over 8-weeks' time through an empirical classification based on the length of clinical distress. In our sample, almost two out of tree (59.4%) HCWs did not manifest moderate or severe distress during the monitoring time. Almost one out of three participants were affected by short-term distress, mostly post-traumatic symptoms that lasted less than weeks. Longer psychological distress occurred only in a smaller percentage (12.6%) of cases, as a combination of more intensive posttraumatic, depressive and anxiety symptoms that lasted for more than 3 weeks on average. Compared with percentages of symptoms estimated by studies with cross-sectional methodologies (3–5), findings from the present study tend to provide a more complex and resiliency-oriented representation of psychological distress, which, although present in 40 percent of HCWs, tends to resolve within a few weeks. At the same time, our findings are in line with stress response studies. In a review conducted on those studies, Galatzer-Levy et al. (10) estimated a prevalence of resilient trajectories of 0.65 (95%CI 0.62–0.70) and a prevalence of chronicity (similar to our longer term distress category) of 0.10 (95%CI 0.09–0.13). The percentage of participants with PTSD symptoms (34.0%) is almost comparable with the range (34.2–57.7%) reported in meta-analysis studies (3–5). However, the percentages of participants with depressive symptoms (22.8%) and anxiety symptoms (22.2%) are lower than aforementioned studies (depressive symptoms = 31.8%−60.5%; anxiety symptoms 34.2%−57.7%).

In addition to estimating the prevalence of the different stress responses, findings allow us to better describe the profiles of distress response in STD and LTD groups. Participants that manifest short term distress experience acute stress reaction in which the interplay between personal, family and professional life events is associated with the stress response. Conversely, longer term distress response in HCWs presents a more complex mental health condition with an higher level of impairment and support needs compared to participants with short-term distress. Compared to STD group, participants with LTD report a high concurrence of PTSD, depressive and anxiety symptoms, an higher prevalence of reported PTSD and traumatic lifetime experiences, higher rates of sick leave due to psychological needs and have most frequently used employee support or mental health professionals in the community. This overlap between PTSD and other distress conditions has already been documented in the literature. According to epidemiological studies, after a traumatic event about 50% of subjects with PTSD also presented a depressive disorder (49–51) and the DSM-5 includes “negative alterations in cognition and mood symptoms” within the set of diagnostic criteria of the PTSD (52). Association between more severe PTSD symptoms and depressive symptoms was also found in HCWs during the COVID-19 emergency in Italy (16). Therefore, it can be said that the long-term distress trajectory identifies a mixed traumatic and depressive response, in which, in addition, the higher recurrence of lifetime depression diagnosis would suggest a recurrent or chronic depressive disorder (53).

Determinants of different types of stress response between resilient, STD and LTD individuals were also identified. Results of the regression models suggest that perceived job stress is the most evident proximal predictor of distress. Stress could elicit a wide range of individual reaction, from short term adjustment to stress-related disorders, such as depression and PTSD (54) and the effect of prolonged work-related stress on workers' physical and mental health is well documented in the occupational health literature (55, 56) and received research focus during the pandemic emergency. Rapisarda et al. (32) developed a model based on two samples of mental health workers (one from Lombardy, Italy, and one from Montreal, Canada) in which work related stressors, including fear of COVID-19, played a role in increasing burnout which, in turn, increases the risk for anxiety-depressive symptoms. Furthermore, by examining the odds ratios of the nominal model, we can see that the predictive effect of perceived stress is different in the two distress groups, where in the LTD group the value is 1.5 times higher than in the STD group: it could be hypothesized that individuals who manifest LTD may have a higher sensitivity to stress compared to resilient and STD individuals.

### 4.1. Limitation, strengths and future research

A first limitation concerns the large rate of participants with incomplete data, especially ones that did not complete the final post-monitoring questionnaire despite they monitored their level of distress with the app. We hypothesize that, during the first and second waves of COVID-19, HCWs were very work loaded and, therefore, filling out a survey could be an additional element of strain that led many participants to avoid extending their participation to this phase as well, as already reported in previous studies (57). Indeed, it should be noted that the completion of this additional survey, which was necessary to collect relevant variables to describe the participants' profiles, was done separately from the app, through an emailed link that referred to an online questionnaire. It is therefore possible to speculate that this change of medium, from the app to the site *via* a link on the email, was a deterrent for many people to continue with the completion of data collection, despite having repeatedly filled out the weekly monitoring questionnaires for several weeks. This reflection suggests that in future studies, we should also try to collect user profiling data through the app and further investigate user experience and usability (58).

A second limitation is that in choosing to operationalize stress response trajectories, only the number of weeks above threshold was considered, leaving out some relevant qualitative information. For example, a participant classified as STD might begin to manifest a clinical level only at the end of 8 weeks, according to a trajectory that previous studies would describe as delayed stress response. It would also be interesting to explore whether, for example, types of distress may alternate over time: if, for example, 1 week one may have only severe anxiety, and in the next week depression but not anxiety. However, we believe that the classification proposed in this study may represent a feasible synthesis that combines the need to provide a limited number of distress response classification categories with the possibility of investigating the determinant and distinguishing characteristics of participants within these subgroups.

A third limitation concerns the selection, the measurement and the modeling of psychological distress determinants. In this study, investigators deployed a set of items that were believed, a priori, to be potential predictors of distress, and an exploratory data analysis was executed. However, although the results identified some relevant variables (such as perceived job stress or the presence of prior mental health problems), there are some issues that may undermine the validity of the results. First, most of the determinants (with the only exclusion of the LEC5 questionnaire) were simple *ad hoc* items (e.g., perceived work stress or perceived support) that may have weaker psychometric properties compared to validated questionnaires. However, this methodological decision was taken to collect, on a weekly base, a wide range of information from participants while maintaining short completion times, which could not have been ensured with validated questionnaires that require groups of items to measure only one construct. Second, the precise relationship network between determinants and distress remains unclear. For example, considering the role of perceived stress, the relationship between it and psychological distress may not necessarily be unidirectional (59); moreover, it may have a mediating effect of some other variables, such as fear of contagion, on psychological distress (32). Therefore, future studies could attempt to better model the reciprocal interactions between variables over time, such as using the technique of path analysis or linear mixed models using time-lagged variables.

Despite these limitations, this study has the merit of being the first of its kind, that is, to have attempted to translate stress response trajectories (10, 14), developed in posttraumatic stress studies, into simple subgroups based on duration of distress, to better estimate and describe the psychological distress of HCWs than has been done in cross sectional research. To do this, the integration of EMA methodology into the longitudinal design provided an opportunity to collect a rich set of information on participants' mental health and experience that mapped the evolution of stress (and some of its determinants) week by week.

We believe that the results of this study may have implications both for research and for design of interventions. Our findings may suggest some practical recommendations that consistent with guidelines on workers' mental health (60, 61) that distinguish between “universal, selective and indicated” interventions according to risk factors and workers' profile: first, universal strategies like stress management interventions and job-design may foster overall mental health; second, HCWs with small children at home and an history of anxiety problems can be considered an at risk group, especially during emergencies, and work-life balance interventions should be promoted to reduce risk of transient but clinically relevant mental health problems; third, HCWs with an history of depressive symptoms should be allow to access specific clinical interventions [like cognitive behavioral therapy (62)] inside or outside the workplace but, at the same time, need to perceive a supportive role form the organization on the whole. At research level, further studies could be designed to replicate the distinction between SDT and LTD in terms of distinctive feature and determinants. This differentiation could be also assessed retrospectively in cross-sectional studies, asking participants about the length of their symptoms. Also, the partial overlap between mental health distress scores [already documented in network studies (20, 63) and dimensional/transdiagnostic approaches (64)] indicates that psychological distress should be assessed using multiple but complementary tools, avoid focusing only on one family of symptoms, like depression or anxiety or post traumatic ones.

Finally, possible future application of these findings involve the development of additional features of distress monitoring apps for smartphones. Adopting a data science approach (65), machine leaning models could be trained to predict clinical profiles such as the ones (RES, STD and LTD) proposed in this study, opening to new research and practical application, such as developing individualized feedback.

## 5. Conclusion

The results of this study, in addition to confirming that resilience is the response of more than half of HCWs to the pandemic emergency, also suggest that those who experience clinical-level distress could be further divided in two groups depending on the length of clinical distress.

The resulting profiles of STD and LTD participants are, at least in part, qualitatively different: people with STD, appear to suffer from stress overload, but have a rapid recovery that impacts work in a limited way; LTD people, by contrast, have a more intense, long, and complex clinical reaction, and the fact that the lifetime presence of depression is a risk factor suggests that this category has a greater propensity for mental health problems, and that intense stress induces relapse.

Based on these differences, different types of interventions can be design and delivered to support workers' mental health that are consistent with WHO recommendations.

## Data availability statement

The raw data supporting the conclusions of this article will be made available by the authors, without undue reservation, by submitting a request to the corresponding author.

## Ethics statement

The studies involving human participants were reviewed and approved by Comité d'éthique á la recherche (CÉR), Centre hospitalier de l'Université de Montréal, Pavillon R, 900, rue Saint-Denis Montréal, QC H2X 0A9, ethique.recherche.chum@ssss.gouv.qc.ca protocol number MP-02-2021-8963, 20.015. The patients/participants provided their written informed consent to participate in this study.

## Author contributions

FR: data analysis and writing—original draft. NB: conceptualization, methodology, and writing—review and editing. M-MD: methodology, data curation, and writing—review and editing. SGu: conceptualization and methodology. SGe: conceptualization, funding acquisition, methodology, supervision, and writing—review and editing. All authors contributed to the article and approved the submitted version.
